# Challenges in using the dynamic components of the SOFA score in health care databases

**DOI:** 10.62675/2965-2774.20240224-en

**Published:** 2024-10-31

**Authors:** Roberta Muriel Longo Roepke, Cornelius Sendagire, David Pilcher

**Affiliations:** 1 Universidade de São Paulo Faculdade de Medicina Hospital das Clínicas São Paulo SP Brazil Trauma and Acute Care Surgery Intensive Care Unit, Hospital das Clínicas, Faculdade de Medicina, Universidade de São Paulo - São Paulo (SP), Brazil.; 2 Makerere University College of Health Sciences Department of Anaesthesia and Critical Care Kampala Uganda Department of Anaesthesia and Critical Care, Makerere University College of Health Sciences - Kampala, Uganda; 3 Alfred Health Department of Intensive Care Melbourne Australia Department of Intensive Care, Alfred Health - Melbourne, Australia

Organ dysfunction and its association with morbidity and mortality in critical illnesses were first described in the 1970s as being closely related to the initial understanding of sepsis pathophysiology. With an increasing understanding of intensive care, we later found that organ failure was a common pathway for multiple etiologies of acute critical illness, such as surgery, trauma, and major burns, which highlighted the need to adequately describe organ failure.

Many scores have been developed to describe organ dysfunction. The Sequential Organ Failure Assessment (SOFA) was developed in 1996 as an easily applicable bedside tool with the purpose of objectively describing organ dysfunction and assessing its severity over time.^(1)^ Its derivation involved variables that were easily available with cutoffs determined by expert opinions and equal weights from 0 to 4 for its six components. It has the advantage of providing a standardized assessment framework across different settings, promoting consistency in clinical practice and research.

However, over time, intensive care medicine has changed considerably, with recent publications highlighting its limitations. For example, the SOFA score focuses solely on acute dysfunction, but the impact of chronic conditions may not be appropriately quantified. Furthermore, the authors hypothesize that the SOFA score's equal weighting of six organ components may not accurately reflect their variable contributions to mortality risk over time and that there are probably associations between different organ dysfunctions.

Lam et al.'s study underscores a much-needed discussion on how to apply the SOFA score in electronic health record (EHR)-based studies.^(2)^ They retrospectively evaluated how each organ domain of the SOFA score was associated with mortality over time, at 24 hours and on Day 7 of intensive care unit (ICU) admission, in patients from two large databases: 4,926 patients from the Medical Information Mart for Intensive Care IV (MIMIC-IV) and 7,871 patients from the eICU Collaborative Research Database (eICU-CRD). They reported that organ dysfunction was not comparable and that its contribution to mortality changed from Day 1 to Day 7. The liver component emerged as the most predictive factor of mortality on Day 1 across both datasets according to the discrete and binary analyses of both cohorts. On Day 7, neurologic dysfunction was the leading predictor in the eICU-CRD cohort, whereas the respiratory and cardiovascular components were the top predictors in the MIMIC-IV cohort. Conversely, in the MIMIC-IV cohort, neurologic dysfunction was the least valuable predictive factor on Day 7. These findings underscore the dynamic nature of organ dysfunction and its impact on patient outcomes, challenging the static approach of the traditional SOFA score.

This begs the following question: is the SOFA score the problem, or is information bias within databases the issue? When assessing the SOFA score prospectively, many modifications are necessary to ascertain the correct weight for each system.^(3)^ However, when an analysis is retrospectively derived from large databases comprising data from EHRs and daily clinical practice information, measurement error and misclassification may impact the findings. This is notably the case with neurological dysfunction. The Glasgow Coma Scale (GCS) is itself a somewhat limited tool for scoring neurological dysfunction, and it is frequently missing from EHRs, especially among sedated patients. The authors imputed a GCS of 15 for all intubated and sedated patients, which is in line with previous studies, but this might have led to an underestimation of the association of neurological dysfunction with mortality since it does not capture the true variability in neurologic status. The impact of interventions such as mechanical ventilation, extracorporeal membrane oxygenation and renal replacement therapy also impacts the weight of each organ component over time. This might explain why renal dysfunction was one of the weakest predictors in both cohorts at Days 1 and 7, given the possible misclassification of renal dysfunction.

Another possible source of bias is the exclusion of patients who were discharged or died before Day 7. Only a minority of ICU admissions are still in the ICU after 7 days, and the mortality of these patients is higher than that of those who have shorter durations of stay in the ICU. In addition, with increasing time in the ICU, preexisting patient factors such as age and comorbidities become progressively more important in determining survival outcomes.^(4)^ Would the results of this study have been different had all patients admitted to the ICU been included? This raises the question of how to derive the SOFA score when death occurs, which is a relevant issue for clinical trials that use this score as an endpoint.^(2)^ In addition, assessing the SOFA score at only two time points (Day 1 and Day 7) may miss critical changes in organ dysfunction that occur between these points. More frequent assessments (e.g., daily SOFA scores or at least 48 hours) could provide a more granular understanding of how organ dysfunction evolves and its impact on mortality.^(5)^

The authors acknowledged that the different results between the cohorts were probably influenced by their populations, given that the MIMIC-IV is a database of a tertiary academic center with more critically ill patients, more comorbidities and a larger portion of patients that meet the Sepsis-3 criteria. In addition, the eICU-CRD and MIMIC-IV databases represent specific patient populations from the United States. The findings may not be directly applicable to other health care settings with different resources and patient demographics. This highlights the importance of a score that is validated in different settings and etiologies of organ dysfunction but also underscores the risk associated with any score when a dataset shift occurs, e.g., when the case mix becomes different for some reason.

The SOFA score remains a valuable tool in critical care medicine. However, clinical practice has changed considerably in recent decades, with shifts toward less invasive monitoring and more widespread use of relatively novel organ supports, such as extracorporeal membrane oxygenation and noninvasive ventilation, new biomarkers and different patterns of drug use and availability.^(6)^ The SOFA 2.0 is currently under development by expert colleagues, who hopefully navigate this progress in the field.^(7)^ The aforementioned revisions may better reflect the dynamic nature of this score and the contribution of individual components during the course of critical illness. However, we need to be aware of the opportunities, benefits and potential risks of EHR-based studies for studying illness severity scores.
