# Supplementary material for: Challenges in using the dynamic components of the SOFA score in health care databases
Source: Crit Care Sci. 2024 Oct 31;36:e20240224en. doi: 10.62675/2965-2774.20240224-en (PMC11634284; doi:10.62675/2965-2774.20240224-en)
Supplement: Translation

# Translation: Desafios no uso dos componentes dinâmicos do escore SOFA em bancos de dados de assistência médica

**DOI:** 10.62675/2965-2774.20240224-pt

**Authors:** Roberta Muriel Longo Roepke, Cornelius Sendagire, David Pilcher

**Affiliations:** 1 São Paulo SP Brasil Unidade de Terapia Intensiva de Trauma e Emergência Cirúrgica, Hospital das Clínicas, Faculdade de Medicina, Universidade de São Paulo - São Paulo (SP), Brasil.; 2 Kampala Uganda Department of Anaesthesia and Critical Care, Makerere University College of Health Sciences - Kampala, Uganda.; 3 Melbourne Austrália Department of Intensive Care, Alfred Health - Melbourne, Austrália.

A disfunção orgânica e sua associação com a morbidade e a mortalidade em doenças críticas foram descritas pela primeira vez na década de 1970 como estreitamente relacionadas à compreensão inicial da fisiopatologia da sepse. Com o aumento da compreensão da terapia intensiva, descobrimos mais tarde que a falência de órgãos era um fator comum a várias etiologias de doenças críticas agudas, como cirurgia, trauma e queimaduras graves, o que evidenciou a necessidade de descrever adequadamente a falência de órgãos.

Desenvolveram-se muitos escores para descrever a disfunção orgânica. O *Sequential Organ Failure Assessment* (SOFA) foi desenvolvido em 1996 como uma ferramenta de fácil aplicação à beira do leito, com o objetivo de descrever objetivamente a disfunção orgânica e avaliar sua gravidade ao longo do tempo.^(1)^ Sua derivação envolveu variáveis prontamente acessíveis, com pontos de corte determinados por opiniões de especialistas e pesos iguais de zero a quatro para seus seis componentes. Essa escala tem a vantagem de fornecer uma estrutura de avaliação padronizada em diferentes ambientes, promovendo a consistência na prática clínica e na pesquisa.

Entretanto, com o passar do tempo, a medicina de terapia intensiva mudou consideravelmente, e publicações recentes destacam suas limitações. Por exemplo, o escore SOFA se concentra apenas na disfunção aguda, mas o impacto das condições crônicas pode não ser quantificado adequadamente. Além disso, os autores levantam a hipótese de que a ponderação igual do escore SOFA de seis componentes de órgãos pode não refletir com precisão suas contribuições variáveis ao risco de mortalidade ao longo do tempo e que, provavelmente, há associações entre diferentes disfunções de órgãos.

Lam et al. ressaltam uma discussão muito necessária sobre como aplicar o escore SOFA em estudos baseados em prontuários médicos eletrônicos (PME).^(2)^ Os autores avaliaram retrospectivamente como cada domínio de órgão do escore SOFA estava associado à mortalidade ao longo do tempo, em 24 horas e no sétimo dia de admissão na unidade de terapia intensiva (UTI), em pacientes de dois grandes bancos de dados: 4.926 pacientes do *Medical Information Mart for Intensive Care IV* (MIMIC-IV) e 7.871 pacientes do *eICU Collaborative Research Database* (eICU-CRD). Eles relataram que a disfunção orgânica não era comparável, e que sua contribuição para a mortalidade mudou do primeiro ao sétimo dia. O componente hepático surgiu como o fator mais preditivo de mortalidade no primeiro dia em ambos os conjuntos de dados, segundo as análises discretas e binárias de ambas as coortes. No sétimo dia, a disfunção neurológica foi o principal fator de previsão na coorte eICU-CRD, enquanto os componentes respiratório e cardiovascular foram os principais fatores de previsão na coorte MIMIC-IV. Por outro lado, na coorte MIMIC-IV, a disfunção neurológica foi o fator preditivo de menor valor no sétimo dia. Esses achados ressaltam a natureza dinâmica da disfunção orgânica e seu impacto nos desfechos de pacientes, desafiando a abordagem estática da SOFA tradicional.

Esse fato suscita a seguinte indagação: o problema é a pontuação SOFA ou o viés de informações nos bancos de dados? Ao avaliar a SOFA prospectivamente, muitas modificações são necessárias para determinar o peso correto para cada sistema.^(3)^ No entanto, em uma análise derivada retrospectivamente de grandes bancos de dados que incluem dados de PMEs e informações da prática clínica diária, o erro de medição e a classificação incorreta podem afetar os resultados. Esse é notavelmente o caso da disfunção neurológica. A escala de coma de Glasgow é, por si só, uma ferramenta um tanto limitada para pontuar a disfunção neurológica e, com frequência, não consta dos PMEs, especialmente entre pacientes sedados. Os autores imputaram uma escala de coma de Glasgow de 15 para todos os pacientes intubados e sedados, que é compatível com estudos anteriores, mas isso pode ter levado a uma subestimação da associação da disfunção neurológica com a mortalidade, pois não capta a verdadeira variabilidade do estado neurológico. O impacto de intervenções como ventilação mecânica, oxigenação por membrana extracorpórea e terapia de substituição renal também impacta o peso de cada componente do órgão ao longo do tempo. Isso pode explicar por que a disfunção renal foi um dos preditores mais fracos em ambas as coortes nos dias 1 e 7, dada a possível classificação incorreta da disfunção renal.

Outra possível fonte de viés é a exclusão de pacientes que receberam alta ou morreram antes do sétimo dia. Apenas uma minoria das admissões na UTI permanece na UTI após 7 dias, e a mortalidade desses pacientes é maior do que a daqueles que têm períodos mais curtos de estadia na UTI. Além disso, com o aumento do tempo na UTI, fatores preexistentes do paciente, como idade e comorbidades, tornam-se progressivamente mais importantes na determinação dos resultados de sobrevivência.^(4)^ Os resultados deste estudo teriam sido diferentes se todos os pacientes admitidos na UTI tivessem sido incluídos? E, novamente, isso levanta a questão de como derivar a pontuação SOFA quando ocorre a morte, o que é uma questão relevante para ensaios clínicos que usam essa pontuação como um desfecho.^(2)^ Além disso, avaliar a pontuação SOFA em apenas dois pontos de tempo (primeiro e sétimo dia) pode perder mudanças críticas na disfunção orgânica que ocorrem entre esses pontos. Avaliações mais frequentes (por exemplo, escores SOFA diários ou pelo menos a cada 48 horas) poderiam fornecer uma compreensão mais granular de como a disfunção orgânica evolui e seu impacto na mortalidade.^(5)^

Os autores reconheceram que os diferentes resultados entre as coortes provavelmente foram influenciados por suas populações, uma vez que o MIMIC-IV é um banco de dados de um centro acadêmico terciário com mais pacientes graves, mais comorbidades e uma parcela maior de pacientes que atendem aos critérios da Sepse-3. Além disso, os bancos de dados do eICU-CRD e do MIMIC-IV representam populações específicas de pacientes dos Estados Unidos. Os achados podem não ser diretamente aplicáveis a outros ambientes de assistência médica com diferentes recursos e dados demográficos dos pacientes. Isso destaca a importância de um escore validado em diferentes ambientes e etiologias de disfunção orgânica, mas também ressalta o risco associado a qualquer escore quando ocorre uma mudança no conjunto de dados, por exemplo, quando a combinação de casos se torna diferente por algum motivo.

O escore SOFA continua sendo uma ferramenta valiosa na medicina de cuidados intensivos. No entanto, a prática clínica mudou consideravelmente nas últimas décadas, com mudanças em direção ao monitoramento menos invasivo e ao uso mais difundido de suportes de órgãos relativamente novos, como a oxigenação por membrana extracorpórea e a ventilação não invasiva, novos biomarcadores e diferentes padrões de uso e disponibilidade de medicamentos.^(6)^ O SOFA 2.0 está sendo desenvolvido atualmente por colegas especialistas, que esperam navegar por esse progresso na área.^(7)^ As revisões mencionadas podem refletir melhor a natureza dinâmica desse escore e a contribuição de componentes individuais durante o curso da doença crítica. No entanto, precisamos estar cientes das oportunidades, dos benefícios e dos possíveis riscos dos estudos embasados em PMEs para investigar os escores de gravidade de doenças.
